# Areas of Agreement and Disagreement Regarding Ponderosa Pine and Mixed Conifer Forest Fire Regimes: A Dialogue with Stevens et al.

**DOI:** 10.1371/journal.pone.0154579

**Published:** 2016-05-19

**Authors:** Dennis C. Odion, Chad T. Hanson, William L. Baker, Dominick A. DellaSala, Mark A. Williams

**Affiliations:** 1 Earth Research Institute, University of California Santa Barbara, Santa Barbara, California, United States of America; 2 Environmental Studies Department, Southern Oregon University, Ashland, Oregon, United States of America; 3 Earth Island Institute, Berkeley, California, United States of America; 4 Program in Ecology and Department of Geography, University of Wyoming, Laramie, Wyoming, United States of America; 5 Geos Institute, Ashland, Oregon, United States of America; 6 Program in Ecology and Department of Geography, University of Wyoming, Laramie, Wyoming, United States of America; Oregon State University, UNITED STATES

## Abstract

In a recent PLOS ONE paper, we conducted an evidence-based analysis of current versus historical fire regimes and concluded that traditionally defined reference conditions of low-severity fire regimes for ponderosa pine (*Pinus ponderosa*) and mixed-conifer forests were incomplete, missing considerable variability in forest structure and fire regimes. Stevens et al. (this issue) agree that high-severity fire was a component of these forests, but disagree that one of the several sources of evidence, stand age from a large number of forest inventory and analysis (FIA) plots across the western USA, support our findings that severe fire played more than a minor role ecologically in these forests. Here we highlight areas of agreement and disagreement about past fire, and analyze the methods Stevens et al. used to assess the FIA stand-age data. We found a major problem with a calculation they used to conclude that the FIA data were not useful for evaluating fire regimes. Their calculation, as well as a narrowing of the definition of high-severity fire from the one we used, leads to a large underestimate of conditions consistent with historical high-severity fire. The FIA stand age data do have limitations but they are consistent with other landscape-inference data sources in supporting a broader paradigm about historical variability of fire in ponderosa and mixed-conifer forests than had been traditionally recognized, as described in our previous PLOS paper.

## Introduction

The accompanying paper by Stevens et al. [1] is critical of one of the several lines of evidence in Odion et al. (2014) [2] that indicate the traditional reference conditions of low-severity fire regimes are incomplete for most ponderosa pine and mixed-conifer forests of western North America. Specifically, Stevens et al. [1] believe that the stand age attribute in Forest Inventory and Analysis (FIA) data is not a useful descriptor of historical fire regimes in ponderosa pine and mixed-conifer forests.

Here, we first briefly summarize points of agreement between Stevens et al. [1] and us, and then discuss in more detail areas where we disagree, including the analysis and interpretation of FIA stand age data. Authorship of this reply is comprised by those who conducted the FIA portion of Odion et al. (2014) [2], as well as authors of Odion et al. whose contributions and backgrounds were needed to respond to FIA-critique elements by Stevens et al. [1] that went beyond the scope of the FIA analysis in Odion et al. (2014) [2].

## Areas of Agreement

### High-severity fire is a natural component of ponderosa pine and mixed-conifer fire regimes

In Odion et al. (2014) [2], we presented several lines of converging evidence that high-severity fire was an important part of historical fire regimes in ponderosa pine and mixed-conifer forests. Over three-quarters of our results pertained to lines of evidence other than FIA stand age data. Stevens et al. [1] reviewed this evidence, some of which was based upon studies published by co-authors of Stevens et al., and concluded the following: “High-severity fire was undoubtedly a component of fire regimes in ponderosa pine and drier mixed-conifer forests.” This represents a significant shift from perspectives in much of the literature in recent decades, which often mentions only low- or low-moderate severity fire in describing historical fire regimes in ponderosa pine and mixed-conifer forests.

### Significant tree recruitment occurs in the absence of fire

We did not intend to suggest that tree recruitment occurred only with fire. Stevens et al. hypothesize that pulsed recruitment in the absence of fire has shaped the age distributions in many FIA plots. We agree that this process occurred historically. There is also agreement that a dominant cohort of trees will establish after high-severity fire, but that later in stand development understory recruitment can happen with favorable climate or following insect outbreaks. This, along with the presence of some trees that pre-date the fire, will create an uneven-aged stand, but there may still be a dominant overstory size class established after fire.

### FIA Stand Age Data May Provide Evidence Consistent with Past High-Severity Fire

Stevens et al. [1] report that 42% of the FIA plots used in Odion et al. (2014) [2] had demographic characteristics consistent with a mortality and recruitment event corresponding generally with the FIA stand age. These plots had an estimated 0–10% of the stand basal area in trees that were older than the stand age. The rest of the basal area (all of it in many cases) was from trees that established after (more recently than) the stand age date, even though most of the plots had stand ages < 200 years old. Despite some qualifications, Stevens et al. [1] conclude that it is plausible that these 42% of plots were visited by historical high-severity fire. However, although Stevens et al. recognize high-severity fire as a component of ponderosa pine and mixed-conifer forests, the definition (threshold of mortality) and patch size of high-severity fire remain a matter of considerable debate.

## Areas of Disagreement

### Appropriate threshold of mortality for high severity fire

Stevens et al. replaced the traditional 70–100% mortality definition for high-severity fire (see, e.g., [3]) that we used with a new 90–100% definition, which means their analysis does not replicate ours and does not refute our findings. Even though this replacement invalidates their analysis of our study, 42% of the FIA plots still have demographic characteristics consistent with high-severity fire with their narrowed definition. Using our original 70–100% mortality definition, there is agreement on 68% of FIA plots regarding demographic characteristics consistent with high-severity fire (and the level of agreement is even higher than this, due to a calculation error in Stevens et al., as discussed below).

Stevens et al. [1] suggest, based on findings of Miller and Quayle (2015) [4], that the high-severity fire definition used by Odion et al. (2014) [2] should be narrowed from 70–100% basal area mortality to 90–100% basal area mortality because Miller and Quayle found that high-severity fire field plots with less than 100% tree mortality were rare. However, 34% of all of their plots with ≥75% basal area mortality had live, surviving trees [4]. Thus, surviving trees in high-severity fire plots were not rare based on data that they cite. Further, Miller and Quayle [4] used plots ranging in size from 0.07 ha to 0.63 ha, while FIA plots consist of four subplots spread over an area of 1.0 ha. Thus, the plots of interest here are more likely to contain surviving trees than those of Miller and Quayle [4]. Further, Miller and Quayle (2015) [4] indicate a user and producer accuracy of 11.1 and 19.2 percent for classifying areas with 75–89% percent basal area mortality. Therefore areas with 75–89% mortality were often not identified correctly in their study.

There is also a logical problem: if high-severity fire predominantly caused 90–100% mortality historically, and 70–89% mortality was rare, then there would be very little difference between the number of FIA plots with 90–100% mortality and the number with 70–100% mortality. But, Stevens et al. found a large difference when using these basal area thresholds. Therefore, plots with 70–89% mortality were not rare, and narrowing the fire-severity definition is not supported.

Stevens et al. [1] state that the “minimum threshold of 70% mortality used by Odion et al. [2] to describe a high-severity patch (and the 75% threshold employed by Landfire) was not developed to describe mortality within a stand, but rather mortality across an entire fire.” However, the two studies cited by Stevens et al. [1] to support this, Agee (1993) [3] and Barrett et al. (2010) [5], say the opposite (see page 23 of Agee 1993 [3], and page 30 of Barrett et al. (2010) [5].

### Plot sizes needed to define high-severity fire

Stevens et al. [1] point out that FIA plot footprints are only 0.4 ha in size in California, Oregon, and Washington, and are only 0.067 ha in size in the other western U.S. states, and use this to suggest that the FIA plots analyzed by Odion et al. [2] were too small to capture true high-severity fire effects. However, Stevens et al. [1] recognize high-severity fire patches as small as 0.4 ha as representing high-severity fire effects. Further, although the total footprint of subplots in FIA plots may be only 0.067 or 0.4 ha, these subplots are representative of a 1.0 ha area. The FIA plots do not capture the size and shape of patches of historical fire, and do not encompass many high-severity patches, which we recognize. But, because they are probabilistic samples, the amount of high-severity fire captured by FIA is a statistical estimate of total amount of high-severity fire. It would be a problem if high-severity fire were rare, or if only a small number of FIA plots were analyzed, but evidence for high-severity fire was abundant, and we analyzed thousands of plots.

### Use of diameter-age relationships for reconstructing past basal area of trees

To understand historical forest structure and fire, it is common to reconstruct the size of trees in the 1800s by subtracting tree growth since that time (e.g., [6]). Stevens et al. recognize that the “basal area of the surviving older trees would have increased in the decades between the year implied by the FIA stand age and the measurement date, thus potentially overestimating their past contribution to the stand basal area in the year implied by the FIA stand age.” In other words, to the extent that the basal area of surviving trees is overestimated, this translates directly to an under-representation of the potential occurrence of historical high-severity fire. However, Stevens et al. [1] did not subtract the basal area that overestimates the past contributions of surviving trees. The effects can be seen via the following general simulation.

Suppose a plot was burned by high-severity fire 100 years ago with 6.1% basal area surviving fire consisting of 16 m^2^ of dead tree basal area. There are 5 live trees of 0.5 m in diameter at breast height (dbh) in the 1-ha FIA plot for a total of 1 m^2^ live, surviving basal area. The surviving trees have a higher growth increment in earlier years which decreases as they age. However, when the mean growth rate is calculated using 1594 mature ponderosa pine in dry forests in Oregon [7], the effects of the slower growth at old age is included to give a mean of 0.45 cm dbh/yr. By not considering the growth rates of surviving trees, surviving basal area at the time of the fire would be overestimated by 3.5 times 100 years later. After two hundred years, the age of some FIA plots, the overestimate would be nearly 8 times the actual plot survivorship, with nearly half the basal area incorrectly considered to have survived since prior to the stand age date. Mortality of mature trees after (more recent than) the stand age date could have occurred in some cases, reducing the overestimates by Stevens et al., but this would likely be a small amount compared to the large magnitude of the overestimates. Thus, the potential effects of high-severity fire were greatly underestimated by Stevens et al.

### Evidence for historical high-severity fire patches >1,000 ha in size

Stevens et al. [1] suggest that high-severity fire patches >1,000 ha in size in some current fires represent a “departure” from historical conditions. However, DellaSala and Hanson (2015) ([8]: pp. 30–33) present numerous examples of historical data sources documenting high-severity fire patches >1,000 ha occurring before fire suppression in previously unlogged forests in both ponderosa pine and mixed-conifer forest types in every major region of the western U.S. Even though large high-severity patches may have been infrequent, they accounted for most high-severity fire [9].

### Combining fire scar data and stand structure data from different plots

Stevens et al. [1] try to test the hypothesis that there would be minimal tree recruitment in the absence of high-severity fire in the FIA plots we studied. However, the locations chosen by Stevens et al. [1] to evaluate recruitment and fire in FIA plots did not actually include any FIA plots. The locations were mostly subjectively selected plots known to not have had severe fire in their long fire-scar history. The plots were up to 1 km away from any FIA plots. Therefore, they do not represent the population of FIA plots we studied.

### Fire and tree recruitment

In all six regions we analyzed in Odion et al., the onset of fire suppression about a century ago coincides with a dramatic reduction in the initiation of trees that form the dominant overstory size classes. Thus, the removal of fire had a profound effect on the process of recruitment over vast areas. Recruitment following fire suppression, as hypothesized by Stevens et al., could not account for the pattern of abundant establishment of the dominant size classes of trees before fire suppression. If high-severity fire was a minor process in creating new stand ages, establishment of the dominant overstory trees would not have declined so dramatically with fire suppression.

### Stevens et al. claim that “Most” ponderosa pine forests and “many” low/mid-elevation mixed-conifer forests historically were “Low-density” forests with frequent, fuel-limited low/moderate-severity fire regimes is not supported by the evidence

This suggestion by Stevens et al. [1] overstates certain evidence, and does not consider other evidence. The sources cited by Stevens et al. [1] are a biased selection of studies that were mostly conducted at relatively local spatial scales, and were often in old-growth forests that are inherently low-density and by definition have not experienced high-severity fire for centuries. The sources cited by Stevens et al. also include studies of current tree densities that try to determine past tree densities but do not have any way to measure historical trees that died, fell, and decayed, and studies where the past effects of logging or fuel wood cutting (when mining occurred and large amounts of wood fuel was needed) cannot be ruled out [2] or where incomplete historical survey data were used [10]. Additionally, Stevens et al. omit reference to dozens of scientific sources indicating more variable historical ponderosa pine and mixed-conifer forests.

In contrast, Odion et al. (2014)[2] reviewed dozens of historical data sources and reconstructions, finding that historical ponderosa pine and mixed-conifer forests: (1) were highly variable in structure/density; (2) had highly variable fire severity, and most forests were dominated by mixed- and high-severity fire; and (3) consistently had a significant component of open forests dominated by low-severity fire at any given time.

## Conclusion

The concern raised by Stevens et al. [1] pertains to only one of the multiple lines of evidence in Odion et al. [2] that together strongly support the historical importance of high-severity fire in ponderosa pine and mixed-conifer forests of the western U.S. Stevens et al.’s comment, specifically on stand age analysis based on Forest Inventory and Analysis field plots, does not refute our study. This is because it is based on a different definition of high-severity fire than the classical definition used by Odion et al. (2014) [2], which is consistent with scientific literature. The new definition proposed by Stevens et al. [1] is based on errors and mischaracterizations of cited sources. Using our definition or theirs of high severity, Stevens et al. [1] found that many FIA plots had demographic structure consistent with a high-severity fire in the 200 years prior to fire suppression and the number of these plots was likely a large underestimate due to the improperly narrow definition of high-severity fire used by Stevens et al., and a major calculation error in their methods.
